# Association of cleft lip and palate on mother-to-infant bonding: a cross-sectional study in the Japan Environment and Children’s Study (JECS)

**DOI:** 10.1186/s12887-019-1877-9

**Published:** 2019-12-20

**Authors:** Shinobu Tsuchiya, Masahiro Tsuchiya, Haruki Momma, Takeyoshi Koseki, Kaoru Igarashi, Ryoichi Nagatomi, Takahiro Arima, Nobuo Yaegashi, Japan Environment & Children’s Study Group, Shin Yamazaki, Yukihiro Ohya, Reiko Kishi, Koichi Hashimoto, Chisato Mori, Shuichi Ito, Zentaro Yamagata, Hidekuni Inadera, Michihiro Kamijima, Takeo Nakayama, Hiroyasu Iso, Masayuki Shima, Youichi Kurozawa, Narufumi Suganuma, Koichi Kusuhara, Takahiko Katoh

**Affiliations:** 1 Department of Orthodontics and Speech Therapy for Craniofacial Anomalies, Tohoku University Hospital, Sendai, Miyagi 980-8574 Japan; 2 Department of Nursing, Tohoku Fukushi University, 6-149-1 Kunimi-ga-oka, Aoba-ku, Sendai, Miyagi 981-3201 Japan; 3 Department of Medicine and Science in Sports and Exercise, Tohoku University Graduate School of Medicine, Sendai, Miyagi 980-8575 Japan; 4 Division of Preventive Dentistry, Tohoku University Graduate School of Dentistry, Sendai, Miyagi 980-8575 Japan; 5 Division of Craniofacial Anomalies, Tohoku University Graduate School of Dentistry, Sendai, Miyagi 980-8575 Japan; 6 Division of Biomedical Engineering for Health & Welfare, Tohoku University Graduate School of Biomedical Engineering, Sendai, Miyagi 980-8575 Japan; 7 Department of Informative Genetics, Environment and Genome Research Center, Tohoku University Graduate School of Medicine, Sendai, Miyagi 980-8575 Japan; 8 Department of Obstetrics and Gynecology, Tohoku University Graduate School of Medicine, Sendai, Miyagi 980-8575 Japan

**Keywords:** Cleft lip and/or palate, Mother-to-infant bonding, Nationwide birth cohort, Psychological distress, Cross-sectional study

## Abstract

**Background:**

Cleft lip and/or palate is among the most prevalent congenital birth defects, and negatively affects maternal psychological status and may consequently result in higher prevalence of child maltreatment. However, the association of childbirths of infants with cleft lip and/or palate with maternal emotional involvement still remains unclear. We examined the association between childbirths of infants with cleft lip and/or palate and mother-to-infant bonding, using data from the Japan Environment and Children’s Study, a nationwide birth cohort study.

**Methods:**

A cross-sectional study using the jecs-an-20,180,131 dataset was performed. A total 104,065 fetuses in 15 regional centres in Japan were enrolled after obtaining informed written consent. The Mother-to-Infant Bonding Scale, a self-report scale consisting of 10 items, was used to evaluate maternal bonding at one year after childbirth. Finally, the participants consisted of 79,140 mother-infant pairs, of which 211 mothers of infants with cleft lip and/or palate were included in our analyses. Multivariable logistic regression analysis using multiple imputation for missing data was performed to calculate the odds ratio and 95% confidence interval in the estimation of the association between bonding disorders and childbirths with cleft lip and/or palate.

**Results:**

No increased risk of bonding disorders was observed among all the mothers of infants with cleft lip and/or palate (odds ratio [95% confidence interval]; 0.97 [0.63–1.48], *p* = 0.880), however, advanced maternal age or multiple parity may adversely affect the associations between bonding disorders and cleft lip and/or palate, respectively. After stratification with a combination of maternal age and parity, a significant association of cleft lip and/or palate with bonding disorders was found only among advanced-age multiparae (odds ratio [95% confidence interval] = 2.51 [1.17–5.37], *p* = 0.018), but it was weakened after additional adjustment for maternal depression.

**Conclusions:**

Childbirths of infants with cleft lip and/or palate may increase the risk of bonding disorders among advanced-age multiparae, possibly through maternal depression. This finding provides valuable information for the provision of multidisciplinary cleft care.

## Background

Cleft lip and/or cleft palate (CL/P), namely cleft lip with or without cleft palate (CL ± P), and isolated cleft palate (CP) are among the most common birth defects and happen at a rate of approximately 1 in 700 births [1]. A nationwide survey in Japan showed that the prevalence of CL/P per 10,000 births was in a range of 14.4–24.8 [1–3], which is higher than the global prevalence. CL/P can be repaired with craniofacial plastic surgeries [2, 4]; however, parents of infants with CL/P generally suffer from parenting and/or caregiving issues as a result of lower infant weight gain due to difficulties in direct breastfeeding and higher risk for upper respiratory infection [5–7]. Mothers of infants with CL/P reportedly tend to show negative moods such as depression and anxiety [8, 9]. Johns et al. found a higher tendency of postpartum depression among older mothers of infants with CL/P [9].

Importantly, Van Horne et al. reported that children with CL/P have higher prevalence rates (7.62% as cumulative probability) of child maltreatment in the U.S. state of Texas, compared with children with congenital diseases such as Down syndrome and spina bifida (approximately 5%) [10]. Most of this maltreatment was in the form of supervisory neglect (about 70%); however, a significantly higher risk of medical neglect was also observed [10, 11]. Boztepe et al. also indicated that, in comparison with congenital heart disease, cleft lip was more likely to adversely affect maternal emotional connection toward the infant possibly due to the visual aspects of the condition [12]. Indeed, there is increasing evidence suggesting potential impairments of infant-maternal relationships among children with CL/P [13–15]. However, the association of childbirths of infants with CL/P on maternal emotional involvement toward infants still remains unclear.

Attachment theory, proposed by Bowlby consists of bidirectional interactions in mother-infant dyads for making children feel secure [16]. In distinction to the infant’s feelings of the attachment, maternal affectionate feelings toward the infant during the perinatal period has been referred to as “mother-to-infant bonding” [17–19]. Bonding disorders, less maternal affection and behaviour toward the infant, have been acknowledged as predictors of impairment in infant development due to child maltreatment [18–21]. Recently, the Mother-to-Infant Bonding Scale (MIBS), which is based on Kumar’s Mother-Infant Bonding Questionnaire [17], has been used for quantitative screening of bonding disorders in mother-infant dyads among the general population [22]. Recent cohort studies, including longitudinal studies, have provided increasingly more evidence [19, 23]. Brockington et al. found that bonding disorders were diagnosed in 29% of mothers with maternal postpartum depression. Indeed, as associated with lifestyle behaviours (drinking and smoking habits) [24], the parity status impacts mother-to-infant bonding because of more requirements regarding maternal attention, especially when a new infant with congenital diseases arrives [25, 26]. Thus, the parity status would confound mother-to-infant bonding with CL/P. Taken together, a better understanding the antecedents of maternal bonding issues after giving birth to a child with CL/P will promote developments in multidisciplinary cleft care.

The aim of this study was to investigate the influence of childbirths of infants with CL/P on bonding disorders using a large-scale sample of the Japan Environment and Children’s Study (JECS), a nationwide, multicentre, prospective birth cohort in Japan and the MIBS.

## Methods

### Study design and participants

**Fig. 1:**
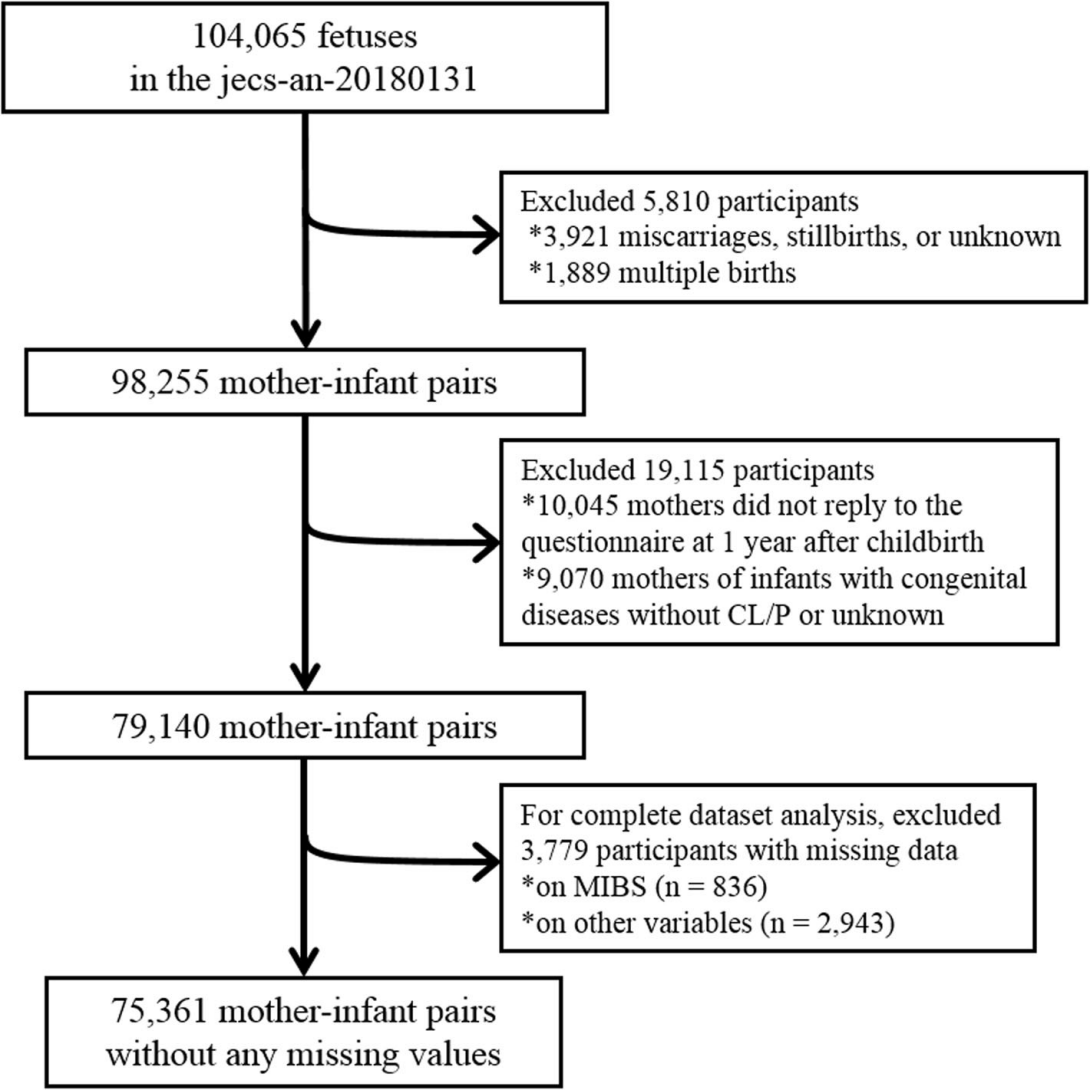
Flow chart of the study participants. MIBS = Mother-to-Infant Bonding Scale

The present study is based on the jecs-an-20,180,131 dataset, which was released in March 2018. In brief, pregnant women in their first trimester were recruited at the first prenatal examination in cooperating hospitals or at local government offices from January 2011 until March 2014. After obtaining informed written consent, participants completed self-administered and medical records/transcripts, and subsequently underwent clinical measurements by medical doctors and trained nurses. To confirm the health status, check-up for both mother and infant was conducted at delivery and one month later. We enrolled 104,065 fetuses in 15 regional centres in JECS. In the fixed data of the JECS, 3921 were miscarriages, stillbirths, and unknown; 1889 were multiple births. Among the 98,255 mother-infant pairs, 10,045 pairs did not reply to the questionnaire sent out at one year after childbirth, and 9070 pairs with other congenital disease(s) without CL/P were excluded from the analysis. A final sample size of 79,140 mother-infant pairs was included in this study (Fig. 1).

### Prevalence of CL/P (exposure measure)

The data on CL/P and other congenital anomalies were ascertained from medical records/transcripts, which were filled by a doctor, a midwife, a nurses or a trained research coordinator at delivery and at one month of age onto JECS transcription forms [3, 27, 28]. The details of data processing, validation, and verification with regards to congenital anomalies were previously described [3]. There are three types of CL/P: cleft lip, cleft palate, or cleft lip with palate. A checkbox for each type was listed on the transcription form. A tick was entered into the corresponding checkbox when any interests of CL/P were observed. Using the fixed JECS dataset, Mezawa et al. reported that total prevalence rates of CL/P per 10,000 births was 24.8 [3].

Furthermore, to examine the influence of visibility of CL/P on the mother-to-infant bonding, the mothers of infants with CL/P were divided into two groups: (1) CL ± P group (mothers of infants with cleft lip with or without cleft palate) and (2) CP group (mothers of infants with isolated cleft palate) as a group with less visible issues.

### Mother-to-infant bonding scale (MIBS: outcome measure)

The MIBS is a self-report scale consisting of 10 items with responses based on a four-point scale (from 0 to 3), and is used to evaluate mother-to-infant bonding at one year after childbirth. The total score ranges from 0 to 30, and higher scores indicate worse mother-to-infant bonding. The MIBS had been translated into Japanese and validated in a previous study [22]. Cronbach’s alpha of the MIBS for the current sample was 0.73. Because the optimal cut-off score is 4/5 [18], the presence of bonding disorders in mother-infant dyads was defined as ≥5 in this study.

### Covariates

In addition to maternal smoking during pregnancy, maternal drinking habits during pregnancy was assessed with a self-administered questionnaire [29]. Maternal age at delivery, parity, and infant sex were ascertained from medical records/transcripts filled by doctors, midwifes, nurses, or trained research coordinators. In a follow-up questionnaire after childbirth, participants also reported feeding pattern and Kessler Psychological Distress Scale scores (K6) at one year after childbirth. The design of the questionnaire has been previously described in detail [27, 28, 30].

Using the data from self-administered and medical records/transcripts, an advanced-age mother was defined as ≥35 years old [31]. In addition, participants were categorized into the following groups by parity (‘primipara’ or ‘multipara’). Smoking status was divided into three categories: ‘never’, ‘stopped smoking before or during pregnancy’, or ‘current smoking’. Alcohol consumption was divided into three categories: ‘never’, ‘stopped drinking’, or ‘current drinking’. Categories for infant sex were ‘male’ or ‘female’, and categories for feeding pattern were ‘breastfeeding’, ‘formula’, or ‘mixed’.

### Statistical analysis

Continuous variables were presented as medians with interquartile ranges, and categorical variables were presented as numbers and percentages (Table 1). With regard to missing data, we applied the ‘missing at random’ assumption, and used multiple imputation with the multivariate normal imputation method [32]. The numbers of participants with missing data in each of the variables are shown in Additional file 1: Table S1. An imputation model including all variables were independently applied for 10 copies of the data, each with missing values suitably imputed. Estimates of the variables were averaged to compute a single mean estimate and adjusted standard errors using Rubin’s rule [33]. We performed crude and multivariate logistic regression analyses using the hierarchical multiple regression model for potential covariates to examine the association of bonding disorders with the prevalence of CL/P within each subgroup. These analyses were performed after adjustment for potential confounding factors, including maternal smoking and drinking habits, feeding pattern, and infant sex (model 1). All parameters in model 1 plus maternal depression (model 2) were included. The OR and 95% CI were calculated for bonding disorders. The results of the multiple imputation analyses are shown in Tables 2 and 3. All statistical analyses were performed using SPSS (version 24.0; IBM Corp., Armonk, NY, USA). In the analysis of the data, *P* values < 0.05 were considered statistically significant.

**Table 1:** Basic characteristics of participating mothers

maternal age	< 35				≥35			
parity	primiparae		multiparae		primiparae		multiparae	
	Healthy	CL/P	Healthy	CL/P	Healthy	CL/P	Healthy	CL/P
	(*n* = 25,628)	(*n* = 71)	(*n* = 31,693)	(*n* = 85)	(*n* = 6536)	(*n* = 18)	(*n* = 15,072)	(*n* = 37)
Age, Median (IQR)	28 (26, 31)	28 (25.7, 31)	30 (28, 32)	30 (27, 33)	37 (36, 39)	37.5 (36, 40)	37 (36, 39)	37 (35.5, 38)
MIBS score, Mean (SD)	2.23 (2.38)	1.75 (1.90)	1.74 (2.20)	2.02 (2.69)	2.21 (2.41)	2.14 (2.57)	1.74 (2.21)	3.08 (3.85)
Bonding disorders (≥5)	3528 (13.8)	5 (7.0)	3104 (9.8)	9 (10.6)	895 (13.7)	3 (16.7)	1503 (10.0)	9 (24.3)
K6 score, Mean (SD)	2.85 (3.63)	3.30 (4.24)	2.86 (3.75)	2.95 (4.15)	2.63 (3.34)	2.22 (2.71)	2.62 (3.39)	3.26 (4.57)
Depression (≥13)	715 (2.8)	2 (2.8)	955 (3.0)	3 (3.5)	128 (2.0)	0 (0)	299 (2.0)	3 (8.1)
Smoking habit, *n* (%)								
Never	15,988 (62.4)	41 (57.7)	17,665 (55.7)	50 (58.8)	3983 (61.0)	12 (66.7)	9153 (60.7)	24 (64.9)
Stopped	8735 (34.1)	27 (38.0)	12,418 (39.2)	30 (35.3)	2379 (36.4)	6 (33.3)	5311 (35.2)	12 (32.4)
Smoking	905 (3.5)	3 (4.2)	1610 (5.1)	5 (5.9)	174 (2.7)	0 (0)	608 (4.0)	1 (2.7)
Alcohol intake, *n* (%)								
Never	8332 (32.5)	26 (36.6)	11,906 (37.6)	31 (36.5)	2009 (30.7)	7 (38.9)	5200 (34.5)	15 (40.5)
Stopped	15,373 (60.0)	42 (59.2)	16,643 (52.5)	43 (50.6)	3894 (59.6)	11 (61.1)	7719 (51.2)	20 (54.1)
Drinking	1923 (7.5)	3 (4.2)	3144 (9.9)	11 (12.9)	633 (9.7)	0 (0)	2153 (14.3)	2 (5.4)
sex, *n* (%)								
Male	12,957 (50.6)	38 (53.5)	16,076 (50.7)	48 (56.5)	3359 (51.4)	11 (61.1)	7809 (51.8)	20 (54.1)
Female	12,671 (49.4)	33 (46.5)	15,617 (49.3)	37 (43.5)	3177 (48.6)	7 (38.9)	7263 (48.2)	17 (45.9)
Feeding pattern, *n* (%)								
Breast	7442 (29.0)	5 (7.0)	11,932 (37.6)	17 (20.0)	1220 (18.7)	0 (0)	5346 (35.5)	5 (13.5)
Mixed	17,670 (68.9)	53 (74.6)	19,008 (60.0)	62 (72.9)	5146 (78.7)	17 (94.4)	9344 (62.0)	26 (70.3)
Formula	516 (2.0)	13 (18.3)	753 (2.4)	6 (7.1)	170 (2.7)	1 (5.6)	382 (2.5)	6 (16.2)

**Table 2:** Association of bonding disorders with the prevalence of CL/P

**< 35, Primiparae,**	**Healthy** (n = 25,628)	**CL/P** (n = 71)	*p* value
Bonding disorders, n (%)	3,528 (13.8)	5 (7.0)	
Crude	1.00	0.51 (0.21-1.26)	0.146
Model 1^a^	1.00	0.44 (0.18-1.09)	0.076
Model 2^b^	1.00	0.44 (0.18-1.12)	0.085
**< 35, Multiparae**	**Healthy** (n = 31,693)	**CL/P** (n = 85)	*p* value
Bonding disorders, n (%)	3,104 (9.8)	9 (10.1)	
Crude	1.00	1.11 (0.55-2.23)	0.780
Model 1^a^	1.00	1.03 (0.51-2.07)	0.946
Model 2^b^	1.00	1.03 (0.50-2.10)	0.941
**≥ 35, Primiparae**	**Healthy** (n = 6,536)	**CL/P** (n = 18)	*p* value
Bonding disorders, n (%)	895 (13.7)	3 (16.7)	
Crude	1.00	1.26 (0.67-2.38)	0.714
Model 1^a^	1.00	1.14 (0.61-2.15)	0.836
Model 2^b^	1.00	1.24 (0.66-2.33)	0.739
**≥ 35, Multiparae**	**Healthy** (n = 15,072)	**CL/P** (n = 37)	*p* value
Bonding disorders, n (%)	1,503 (10.0)	9 (24.3)	
Crude	1.00	2.90 (1.58-5.34)	**0.006**
Model 1^a^	1.00	2.51 (1.17-5.37)	**0.018**
Model 2^b^	1.00	2.18 (0.96-4.95)	0.062

Odds ratio (95% confidence interval) (all such values) for bonding disorders were compered with the reference participants

^a^Adjusted for maternal factors (smoking and drinking habits, feeding pattern, and infant sex)

^b^Additionally adjusted for maternal depression with Model 1

*P* values representing significant differences (<0.05) are indicated in bold

**Table 3:** Association of bonding disorders with the prevalence of cleft lip among advanced-age multiparae

≥35, Multiparae	Healthy (*n* = 15,072)	CL ± P (*n* = 29)	*p* value	CP (*n* = 8)	*p* value
Bonding Disorders, *n* (%)	1503 (10.0)	7 (24.1)		2 (25.0)	
Crude	1.00	2.87 (1.86–4.44)	**0.015**	3.01 (1.33–6.81)	0.178
Model 1^a^	1.00	2.56 (1.07–6.10)	**0.033**	2.36 (0.46–11.95)	0.308
Model 2^b^	1.00	2.31 (0.93–5.73)	0.072	1.76 (0.28–10.93)	0.545

Odds ratio (95% confidence interval) (all such values) for bonding disorders were compared with the reference participants

^a^Adjusted for maternal factors (smoking and drinking habits, feeding pattern, and infant sex)

^b^Additionally adjusted for maternal depression with Model 1

P values representing significant differences (< 0.05) are indicated in bold

## Results

### Participants’ baseline characteristics

The median age of the participants was 31 years (interquartile range: 28–35 years), and the mean MIBS and K6 scores were 1.94 (standard deviation [SD]: 2.29) and 2.79 (SD: 3.61), respectively (Additional file 1: Table S1). The total numbers (%) of infants born with cleft lip with or without palate or isolated cleft palate in the present study were 64 (0.08), 90 (0.11), and 57 (0.07), respectively. Interestingly, the mean maternal MIBS scores (SD) of dyads with infants with CL/P were similar to those of the healthy infants (2.13 [2.72] vs. 1.94 [2.29] in Additional file 1: Table S1), but only advanced-age multiparae with infants with CL/P at birth showed higher MIBS scores (SD) compared with healthy infants (3.08 [3.85] vs. 1.74 [2.21]) as well as prevalence of maternal depression (8.1% vs. 2.0%), as shown in Table 1.

### Association between bonding disorders and CL/P

No risk of bonding disorders was observed among all the mothers with infants with CL/P (odds ratio [OR] [95% confidence interval (CI)] = 0.97 [0.63–1.48], *p* = 0.880). After simple stratification by advanced maternal age or parity (Additional file 2: Table S2), ORs of association between bonding disorders and CL/P in multivariate logistic regression analyses tended to be decreased in mothers aged < 35 (OR [95% CI] = 0.71 [0.40–1.24], *p* = 0.222) or primiparae (OR [95% CI] = 0.58 [0.28–1.22], *p* = 0.152), while they tended to be increased in mothers aged ≥35 (OR [95% CI] = 1.81 [0.91–3.61], *p* = 0.086) or multiparae (OR [95% CI] = 1.39 [0.82–2.35], p = 0.222). Thus, the dataset was used after stratification with a combination of maternal age and parity. The characteristics of participants after stratification are shown in Table 1.

In multivariate logistic regression analysis using the imputed dataset, the adjusted ORs (95% CI) for bonding disorders of mothers of infants with CL/P in each group are summarized in Table 2. Compared with the reference participants with healthy infants, analyses without adjustments (crude model) or adjusted for all covariates except for maternal depression (model 1) revealed that the prevalence of bonding disorders was significantly associated with having an infant with CL/P only in the advanced-age multiparae group (OR [95% CI] = 2.51 [1.17–5.37], *p* = 0.018), but not in the other groups (OR [95% CI]: 0.44 [0.18–1.09], *p* = 0.076 in younger primiparae; 1.03 [0.51–2.07], *p* = 0.946 in younger multiparae; and 1.14 [0.61–2.15], *p* = 0.836 in advanced-age primiparae, respectively, in Table 2). However, additional adjustment for maternal depression (model 2) weakened the statistical association and resulted in no significance between bonding disorders and CL/P among advanced-age multiparae (OR [95% CI] = 2.18 [0.96–4.95], *p* = 0.062). Interestingly, though not significant, CL/P tended to be negatively associated with bonding disorders only among young primiparae (OR [95% CI] = 0.44 [0.18–1.12], *p* = 0.085). Furthermore, analyses of the complete dataset (*n* = 75,361), excluding cases with missing values, also indicated significant association between bonding disorders and CL/P in crude model (OR [95% CI]; 2.39 [1.04–5.51], *p* = 0.040), but not in other models as shown in Additional file 3: Table S3. There were no significant interaction terms in the model between advanced maternal age and parity.

### Association between bonding disorders and cleft lip prevalence

With respect to the association of bonding disorders with the visibility of cleft lip, the prevalence of bonding disorders among advanced-age multiparae was significantly associated with CL ± P in the crude model (OR [95% CI]; 2.87 [1.86–4.44], *p* = 0.015) or model 1 (OR [95% CI]; 2.56 [1.07–6.10], *p* = 0.033), but not in adjusted model for all covariates (OR [95% CI]; 2.31 [0.93–5.73], *p* = 0.072) as shown in Table 3. CP, a group with less visible issues than CL ± P did not also have any significant association with bonding disorders (OR [95% CI]; 1.76 [0.28–10.93], *p* = 0.545). In addition, there were no significant associations between bonding disorders with CL ± P or CP in the other three groups (younger primiparae, younger multiparae. or advanced-age primiparae).

## Discussion

Our present results using the nationwide data from a large-scale birth cohort study in Japan showed no significant association between maternal bonding disorders and CL/P among all the participants (OR [95% CI]; 0.97 [0.63–1.48], *p* = 0.880). However, our finding revealing the significant association of CL/P with maternal bonding disorders among advanced-age multiparae may serve as valuable information for multidisciplinary cleft care providers in terms of the practical benefits of the MIBS in screening for maternal bonding issues.

To the best of our knowledge, this is the first report showing the association of CL/P with mother-to-infant bonding, though only among advanced-age multiparae. Maternal depression, which has been acknowledged as a predictor for bonding disorders [23, 24], statistically impacts the association of maternal bonding disorders with CL/P, because mothers of infants with CL/P are generally troubled with more parenting and/or caregiving issues with regard to feeding and breathing developments [5–7]. Furthermore, because the visual impacts of cleft lip possibly influence the processing of maternal-to-infant bonding as suggested by Boztepe et al. [12], we focused on whether the prevalence of cleft lip was associated with bonding disorders among advanced-age multiparae. Consequently, the significant association of bonding disorders with prevalence of CL ± P did not remain after the adjustment using all covariates (OR [95% CI] = 2.31 [0.93–5.73], *p* = 0.072). This finding may be due to the smaller sample size of mothers having infants with CL/P. Further examination of the confounding effects by visibility of cleft lip in future studies with the appropriate design is warranted.

Our results indicated that the association between bonding disorders and CL/P strongly varies according to parity and maternal age at delivery. Similar to the increasing trends of advanced maternal age and multiple parity on the association between bonding disorders and CL/P (Additional file 2: Table S2), their combined stratification showed a significant association between bonding disorders and CL/P among advanced-age multiparae. A review of relevant studies indicated that the impacts of advanced maternal age and/or parity on mother-to-infant bonding are under some debate; however, several studies have reported adverse effects of older maternal age and multiparity on mother-to-infant bonding [34–37]. Older mothers generally experience a more severe delivery and have more issues regarding parenting due to physical and psychological limitations [38, 39]. Meanwhile, as shown in our results (Table 1), multiparity generally contributes to better mother-to-infant bonding [40, 41]. Therefore, we speculated that the significant association CL/P and bonding disorders among between multiparous mothers may be related to the presence of healthy siblings. It should be noted that the recurrence rate of nonsyndromic CL/P among siblings is reportedly low, 3.2–9.1% [42, 43]. Similarly, Van Horne et al. reported that child maltreatment among children with CL/P increases as the number of siblings increases [11]. Tanimura et al., using nationwide data in Japan, pointed out that sibling comparison by parents (potentially including caregiving with congenital anomalies) may be a common risk factor for child maltreatment [44]. Differences in caregiving among siblings adversely impact maternal feelings and behaviours toward infants with CL/P, whose care typically involves more daily-life stressors [45], and may lead to maternal bonding issues. In order to further examine these findings, careful longitudinal observations among mothers of infants with CL/P are necessary because there may be potential impacts on the children’s development with CL/P from maternal feelings and/or behaviours, even when the mother is giving birth to a new healthy sibling.

This study has several strengths and limitations. Since the Japanese nationwide survey covered approximately 45% of infants born in multi-subject area during 2013, our results, mostly based on the Japanese general population, allowed us to compare the experimental participants with abundant controls [27]. In terms of study limitations, first, this was a cross-sectional study using a one-time measurement of mother-to-infant bonding as the outcome. Future longitudinal studies with more appropriate designs that consider episodes of child maltreatment are warranted. Second, this study’s data collection methods did not include a query about antenatal diagnosis and/or screening. Johns et al. suggested that receiving antenatal diagnosis decreases maternal depressive symptoms among mothers of infants with CL/P [9]. Thus, our findings may be limited because the possibility of artificial abortion related to congenital anomaly after antenatal diagnosis as a selection bias cannot be ruled out.

## Conclusions

This cross-sectional study using Japanese nationwide data indicated that mothers of infants with CL/P had similar rates of bonding disorders as the general population; however, advanced-age multiparae had a significantly higher risk of bonding disorders, and the MIBS may be useful in understanding antecedents of their bonding issues.

## Supplementary information

**Additional file 1: Table S1.** Total basic characteristics of participating mothers

**Additional file 2: Table S2.** Association of bonding disorders with the prevalence of CL/P with stratification either by maternal age or by parity

**Additional file 3: Table S3.** Association of bonding disorders with CL/P among advanced-age multiparae in complete dataset analysis

## Data Availability

The JECS data are not publicly available due to ethical restrictions and the legal framework of Japan. All inquiries about access to the data should be sent to the JECS Programme Office, National Institute for Environmental Studies (jecs-en@nies.go.jp).

## Abbreviations

CI: Confidence interval
CL ± P: Cleft lip with or without cleft palate
CL/P: Cleft lip and/or cleft palate
CP: Isolated cleft palate
IQR: Interquartile range
JECS: Japan Environment and Children’s Study
K6: Kessler Psychological Distress Scale scores
MIBS: Mother-to-Infant Bonding Scale
OR: Odds ratio
SD: Standard deviation
