# Supplementary material for: Association of cleft lip and palate on mother-to-infant bonding: a cross-sectional study in the Japan Environment and Children’s Study (JECS)
Source: BMC Pediatr. 2019 Dec 20;19:505. doi: 10.1186/s12887-019-1877-9 (PMC6923825; doi:10.1186/s12887-019-1877-9)
Supplement: Supplementary file 1 — Additional file 1: Table S1. Total basic characteristics of participating mothers [file 12887_2019_1877_MOESM1_ESM.pdf]

Supplementary Table 1. Total basic characteristics of participanting mothers.

	**Total** (n = 79,140)	**Healthy** (n = 78,929)	**CL/P** (n = 211)
Age, Median (IQR)	31 (28, 35)	31 (28, 35)	31 (27,35)
MIBS score, Mean (SD)	1.94 (2.29)	1.94 (2.29)	2.13 (2.72)
**Bonding disorders (≥5)**	9,056 (11.4)	9,029 (11.4)	27 (12.8)
K6 score, Mean (SD)	2.79 (3.61)	2.79 (3.61)	3.06 (4.14)
**Depression (≥13)**	2,106 (2.7)	2,098 (2.7)	8 (3.8)
Parity, n (%)			
**Primiparae**	32,253 (40.8)	32,164 (40.8)	89 (42.2)
**Multiparae**	46,887 (59.2)	46,765 (59.2)	122 (57.8)
Smoking habit, n (%)			
**Never**	46,915 (59.3)	46,789 (59.3)	126 (59.7)
**Stopped**	28,919 (36.5)	28,843 (36.5)	76 (36.0)
**Smoking**	3,306 (4.2)	3,297 (4.2)	9 (4.3)
Alcohol intake, n (%)			
**Never**	27,526 (34.8)	27,447 (34.8)	79 (37.4)
**Stopped**	43,745 (55.3)	43,629 (55.3)	116 (55.0)
**Drinking**	7,869 (9.9)	7,853 (9.9)	16 (7.6)
Infant sex, n (%)			
**Male**	40,318 (50.9)	40,201 (50.9)	117 (55.5)
**Female**	38,822(49.1)	38,728 (49.1)	94 (44.5)
Feeding pattern, n (%)			
**Breast**	25,966 (32.8)	25,939 (32.9)	27 (12.8)
**Mixed**	51,326 (64.9)	51,168 (64.8)	158 (74.9)
**Formula**	1,848 (2.3)	1,822 (2.3)	26 (12.3)

IQR=interquartile range; SD=standard deviation.

Percentages and numbers of healthy infants and infants with CL/P may not sum to 100 or total numbers owing to rounding.
