# Supplementary material for: Association of cleft lip and palate on mother-to-infant bonding: a cross-sectional study in the Japan Environment and Children’s Study (JECS)
Source: BMC Pediatr. 2019 Dec 20;19:505. doi: 10.1186/s12887-019-1877-9 (PMC6923825; doi:10.1186/s12887-019-1877-9)
Supplement: Supplementary file 2 — Additional file 2: Table S2. Association of bonding disorders with the prevalence of CL/P with stratification either by maternal age or by parity [file 12887_2019_1877_MOESM2_ESM.pdf]

Supplementary Table 2. Association of bonding disorders with the prevalence of CL/P with stratification either by maternal age or by parity.

Total

	Healthy (n =78,929)	CL/P (n=211)	p value
Bonding Disorders, n (%)	9,029 (11.4)	27 (12.8)	
Crude	1.00	1.11 (0.74-1.68)	0.613
Model 1[a]	1.00	0.99 (0.66-1.50)	0.961
Model 2[b]	1.00	0.97 (0.63-1.48)	0.880

Age stratification

**<35**	Healthy (n = 57,321)	CL/P (n = 156)	p value
Bonding Disorders, n (%)	6,632(11.6)	14 (9.0)	
Crude	1.00	0.78 (0.45-1.35)	0.381
Model 1[a]	1.00	0.70 (0.40-1.21)	0.204
Model 2[b]	1.00	0.71 (0.40-1.24)	0.222
**≥35**	Healthy (n = 21,608)	CL/P (n = 55)	p value
Bonding Disorders, n (%)	2,398 (11.1)	12 (21.8)	
Crude	1.00	2.24 (1.61-3.10)	**0.014**
Model 1[a]	1.00	1.95 (1.02-3.72)	**0.043**
Model 2[b]	1.00	1.81 (0.91-3.61)	0.086

Parity stratification

**Primiparae**	Healthy (n = 32,164)	CL/P (n = 89)	p value
Bonding Disorders, n (%)	4,423 (13.8)	8 (9.0)	
Crude	1.00	0.65 (0.32-1.34)	0.244
Model 1[a]	1.00	0.56 (0.27-1.17)	0.122
Model 2[b]	1.00	0.58 (0.28-1.22)	0.152
**Multiparae**	Healthy (n = 46,765)	CL/P (n = 122)	p value
Bonding Disorders, n (%)	4,607 (9.9)	18 (14.8)	
Crude	1.00	1.60 (0.96-2.65)	0.070
Model 1[a]	1.00	1.46 (0.88-2.42)	0.145
Model 2[b]	1.00	1.39 (0.82-2.35)	0.222

Odds ratio (95% confidence interval) (all such values) for bonding disorders were compared with the reference participants.

[a]Adjusted for maternal factors (smoking and drinking habits, feeding pattern, and infant sex).

[b]Additionally adjusted for maternal depression with Model 1.

P values representing significant differences (<0.05) are indicated in bold.
