# Supplementary material for: Association of cleft lip and palate on mother-to-infant bonding: a cross-sectional study in the Japan Environment and Children’s Study (JECS)
Source: BMC Pediatr. 2019 Dec 20;19:505. doi: 10.1186/s12887-019-1877-9 (PMC6923825; doi:10.1186/s12887-019-1877-9)
Supplement: Supplementary file 3 — Additional file 3: Table S3. Association of bonding disorders with CL/P among advanced-age multiparae in complete dataset analysis [file 12887_2019_1877_MOESM3_ESM.pdf]

**Supplementary Table 3. Association of bonding disorders with CL/P among advanced-age multiparae in complete data set analysis.**

| **<35, Primiparae,** | **Healthy** (n = 24,341) | **CL/P** (n = 65) | p value |
|---|---|---|---|
| Bonding disorders, n (%) | 3,295 (13.5) | 4 (6.2) | |
| Crude | 1.00 | 0.42 (0.15-1.15) | 0.092 |
| Model 1[a] | 1.00 | 0.36 (0.13-0.99) | **0.047** |
| Model 2[b] | 1.00 | 0.38 (0.14-1.07) | 0.067 |
| **<35, Multiparae** | **Healthy** (n = 30,051) | **CL/P** (n = 79) | p value |
| Bonding disorders, n (%) | 2,890 (9.6) | 8 (10.1) | |
| Crude | 1.00 | 1.06 (0.51-2.20) | 0.878 |
| Model 1[a] | 1.00 | 0.98 (0.47-2.04) | 0.955 |
| Model 2[b] | 1.00 | 0.97 (0.46-2.05) | 0.939 |
| **≥35, Primiparae** | **Healthy** (n = 6,248) | **CL/P** (n = 17) | p value |
| Bonding disorders, n (%) | 856 (13.7) | 2 (11.8) | |
| Crude | 1.00 | 0.84 (0.19-3.68) | 0.817 |
| Model 1[a] | 1.00 | 0.76 (0.17-3.32) | 0.710 |
| Model 2[b] | 1.00 | 0.82 (0.19-3.59) | 0.790 |
| **≥35, Multiparae** | **Healthy** (n = 14,526) | **CL/P** (n = 34) | p value |
| Bonding disorders, n (%) | 1,420 (9.8) | 7 (20.6) | |
| Crude | 1.00 | 2.39 (1.04-5.51) | **0.040** |
| Model 1[a] | 1.00 | 2.09 (0.90-4.85) | 0.087 |
| Model 2[b] | 1.00 | 2.10 (0.88-5.01) | 0.095 |

Odds ratio (95% confidence interval) (all such values) for bonding disorders were compared with the reference participants.

[a]Adjusted for maternal factors (smoking and drinking habits, feeding pattern, and infant sex).

[b]Additionally adjusted for maternal depression with Model 1.

P values representing significant differences (<0.05) are indicated in bold.
